# Case Report: A rare adult case of *ELANE*-related severe congenital neutropenia revealed by neutropenic enterocolitis

**DOI:** 10.3389/fimmu.2026.1881387

**Published:** 2026-07-08

**Authors:** Zhenhua Jiang, Diya Wen, Yuanyuan Liu, Mingyue Shi, Qiuyu Liu, Yanliang Bai

**Affiliations:** 1 Department of Gastroenterology, Zhengzhou University People’s Hospital and Henan Provincial People’s Hospital, Zhengzhou, China; 2 Department of Hematology, Henan University People’s Hospital and Henan Provincial People’s Hospital, Zhengzhou, China; 3 Department of Hematology, Zhengzhou University People’s Hospital and Henan Provincial People’s Hospital, Zhengzhou, China; 4 Department of Pathology, Zhengzhou University People’s Hospital and Henan Provincial People’s Hospital, Zhengzhou, China

**Keywords:** case report, *ELANE* mutation, Kostmann syndrome, neutropenic enterocolitis, severe congenital neutropenia

## Abstract

Severe congenital neutropenia (SCN) is a rare hematologic disorder characterized by recurrent and potentially life-threatening infections beginning in early childhood, associated with absolute neutrophil counts (ANCs) below 0.5×10^9^/L. Mutations in the *ELANE* gene are the most prevalent cause of SCN. Gastrointestinal complications such as neutropenic enterocolitis (NE) are rarely reported as presenting manifestations of congenital neutropenia. Herein, we report an 18-year-old male with a lifelong history of recurrent oral ulcers, otitis, and skin infections, who presented with intermittent abdominal pain. Computed tomography demonstrated diffuse bowel wall thickening, and colonoscopy revealed a colonic stricture. Due to progressive obstructive symptoms, the patient underwent a laparoscopic right hemicolectomy. The preliminary histopathological analysis indicated a diagnosis of ulcerative colitis (UC). However, persistent severe neutropenia following surgery raised suspicion of an alternative underlying etiology. Subsequent whole-exome sequencing identified a heterozygous likely pathogenic variant in the *ELANE* gene, establishing the diagnosis of SCN. Retrospective clinicopathological reassessment indicated that the gastrointestinal manifestations were more consistent with NE secondary to chronic neutropenia, rather than primary inflammatory bowel disease. To our knowledge, this is among the first reported cases of *ELANE*-related SCN presenting predominantly as NE. This case expands the clinical spectrum of SCN and highlights the importance of considering congenital neutropenia in patients with unexplained gastrointestinal inflammation accompanied by persistent neutropenia, to avoid misdiagnosis and optimize management.

## 1 Introduction

Severe congenital neutropenia (SCN), also known as Kostmann syndrome, is a rare genetic disorder affecting granulopoiesis, characterized by a lack of mature neutrophils in the bone marrow and peripheral blood, with absolute neutrophil counts (ANCs) typically below 0.5×10^9^/L (1). The estimated prevalence ranges from 3 to 8.5 cases per million individuals, and the condition is usually diagnosed in early childhood (1).

Patients with SCN are highly susceptible to recurrent and severe infections beginning in infancy, including otitis, gingivitis, skin infections, pneumonia, deep abscesses, and septicemia, and have an increased risk of progression to leukemia (2). More than half of SCN cases have mutations in the neutrophil elastase, encoded by the *ELANE* gene. These mutations cause the arrest of myelopoiesis at the promyelocyte stage in SCN patients. In addition, *ELANE* mutations can also lead to cyclic neutropenia (CyN), with a 21-day cycle (3). Patients with SCN typically have lower ANCs and more severe infections than those with CyN (4). Beyond recurrent infections, gastrointestinal manifestations have also been reported in congenital neutropenia syndromes, including chronic diarrhea and abdominal pain that resemble inflammatory bowel disease (IBD) (5, 6).

Neutropenic enterocolitis (NE), also known as typhlitis, is associated with high mortality and primarily manifests in immunocompromised patients, especially those receiving cytotoxic chemotherapy (7). It is characterized by bowel wall edema, thickening, ulceration, and bleeding. Clinically, NE often presents with the triad of neutropenia, fever, and abdominal pain. Although the pathogenesis of NE is not fully understood, it is believed to be multifactorial (8, 9). The exact incidence and prevalence of NE are unknown and vary widely from 0.22% to 46% across studies (7).

NE in congenital neutropenia is extremely rare and may be underrecognized or misdiagnosed as inflammatory bowel disease or infectious colitis (10), posing a significant diagnostic challenge, especially when neutropenia is longstanding but unrecognized. While *ELANE-*related SCN has been widely reported, its association with NE has rarely been described. Herein, we report a case of *ELANE*-associated SCN initially presenting as NE and misdiagnosed as IBD. Our report suggests a possible association between *ELANE*-related SCN and NE, thereby broadening the disease spectrum.

## 2 Case description

**Figure 1:**
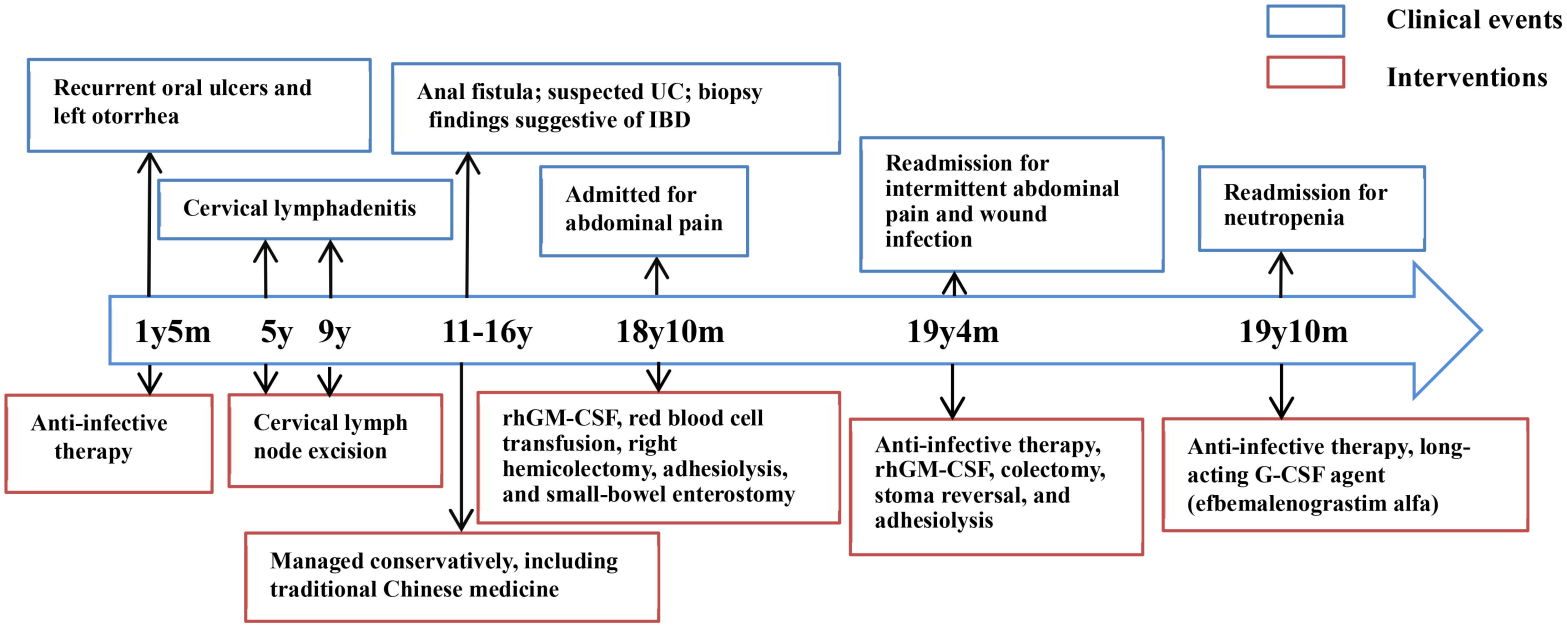
Clinical timeline of the patient.

On October 4, 2024, an 18-year-old man presented to our hospital, in the Gastrointestinal Surgery Department, with intermittent abdominal pain that had progressively worsened over the past two months. He had a history of recurrent infections since early childhood. Shortly after birth, he was diagnosed with neutropenia and cellular immunodeficiency disease in another hospital due to oral ulcers and discharge of pus from the left external ear canal. Over the years, he suffered from recurrent oral ulcers and infections involving the eyes, nose, and scalp, leading to multiple hospitalizations. At the ages of 5 and 9, he underwent bilateral cervical lymph node excision for lymphadenitis. At the age of 11, he was hospitalized for treatment of an anal fistula. At the age of 13, he was considered to have ulcerative colitis (UC) at an outside hospital, although the original diagnostic records were difficult to access. At the age of 16, gastrointestinal endoscopy with biopsy suggested IBD, but the definitive subtype could not be established (**Figure 1**; **Table 1**).

**Table 1:** Serial laboratory findings during selected hospitalizations and follow-up.

Timepoint	WBC count (×10^9^/L)	ANC (×10^9^/L)	Hb (g/L)	Platelet count (×10^9^/L)	Clinical status	Treatment
At index admission (age 18 years 10 months)	1.86	0.55	86	343	Abdominal pain and anemia	rhGM-CSF, red blood cell transfusion, right hemicolectomy, adhesiolysis, and small-bowel enterostomy
Hospital day 7	0.99	0.15	113	326	Neutropenia	rhGM-CSF, anti-infective therapy, and red blood cell transfusion
Hospital day 14	13.94	1.27	97	167	Fever and poor wound healing	Anti-infective therapy, repeated wound care, and VSD
At discharge after index admission	2.68	0.41	77	213	Clinical symptoms and signs improved	Anti-infective therapy and rhGM-CSF
Readmission (2025-03-16)	1.44	0.34	114	317	Intermittent abdominal pain and wound infection	Anti-infective therapy, rhGM-CSF, colectomy, stoma reversal, and adhesiolysis
Readmission (2025-09-22)	2.16	0.25	101	267	Intermittent abdominal pain and neutropenia	Anti-infective therapy and long-acting G-CSF agent (efbemalenograstim alfa)
At discharge (2025-09-27)	3.25	0.37	95	203	Further clinical improvement	Anti-infective therapy

IBD, inflammatory bowel disease; rhGM-CSF, recombinant human granulocyte-macrophage colony-stimulating factor; G-CSF, granulocyte colony-stimulating factor. WBC, white blood cell; ANC, absolute neutrophil count; Hb, hemoglobin; VSD, vacuum sealing drainage.

On the initial physical examination, the abdomen was distended, with tenderness and guarding, most pronounced in the right lower quadrant (RLQ). No superficial lymph nodes or hepatosplenomegaly were palpated. Bowel sounds were hyperactive at 8/min. The remainder of the examination was unremarkable. Laboratory investigations on admission showed a total white blood cell count of 1.86×10^9^/L, an ANC of 0.55×10^9^/L (reference: 1.8–6.3×10^9^/L), a hemoglobin level of 86 g/L, and a platelet count of 343×10^9^/L. The fecal occult blood test was positive.

**Figure 2:**
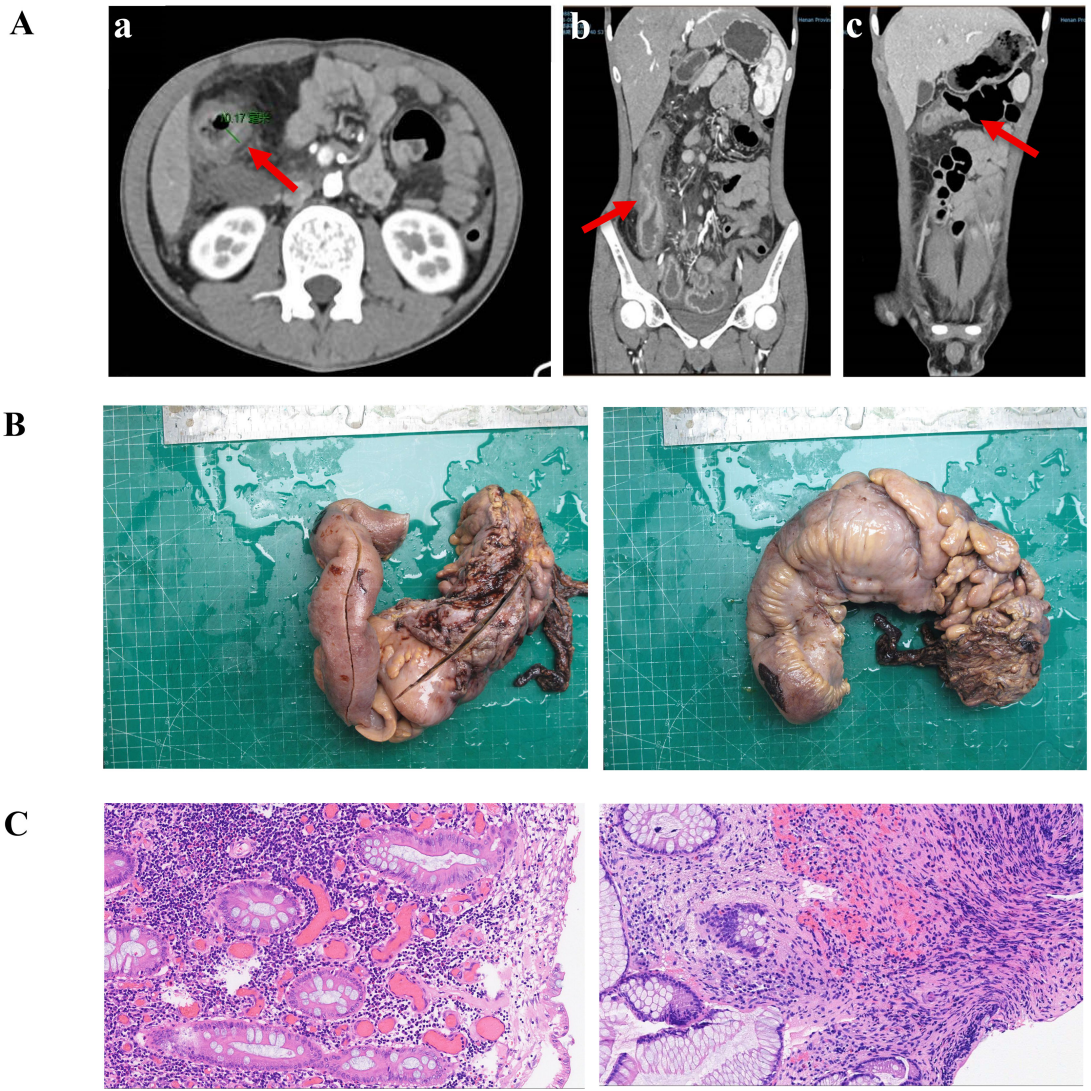
**(A)** CT scan of the patient at the onset of abdominal pain: (a) transverse view of the ascending colon with a wall thickness measuring 10.17 mm as indicated by a red arrow, (b) coronal view of the ascending colon, (c) sagittal view of the ascending colon. **(B)** Ileocecal specimen from laparoscopic exploration, showing marked inflammatory thickening of the intestinal wall. **(C)** Histopathological examination of ileocecal specimen (HE staining).

A computed tomography (CT) scan of the abdomen revealed diffuse thickening and edema of the distal ileum, ileocecal region, ascending colon, and hepatic flexure, with inflammatory exudation (**Figure 2A**). Colonoscopy could not be advanced beyond the hepatic flexure due to a marked luminal stricture. The surrounding mucosa appeared coarse with converging folds, and the lesion could not be clearly visualized, limiting biopsy acquisition. The endoscopic impressions were colonic stricture with colitis.

Due to progressive obstructive symptoms and failure of conservative management, the patient underwent laparoscopic surgical exploration. Intraoperative findings revealed a firm mass extending from the ileocecal region to the hepatic flexure, with inflammatory, dilated, edematous, and rigid changes of the right colon (**Figure 2B**). Right hemicolectomy and intestinal adhesiolysis with small intestine enterostomy were performed.

Histopathologic examination of the resected specimen demonstrated chronic active colitis with crypt architectural distortion, cryptitis, goblet cell depletion, focal reduction in crypt density, inflammatory polyps, and focal ulceration extending into the muscularis propria. In addition, the bowel wall demonstrated marked thickening with fibrosis and chronic inflammatory infiltration, predominantly composed of lymphocytes. These findings were initially interpreted as compatible with UC (**Figure 2C**).

**Figure 3:**
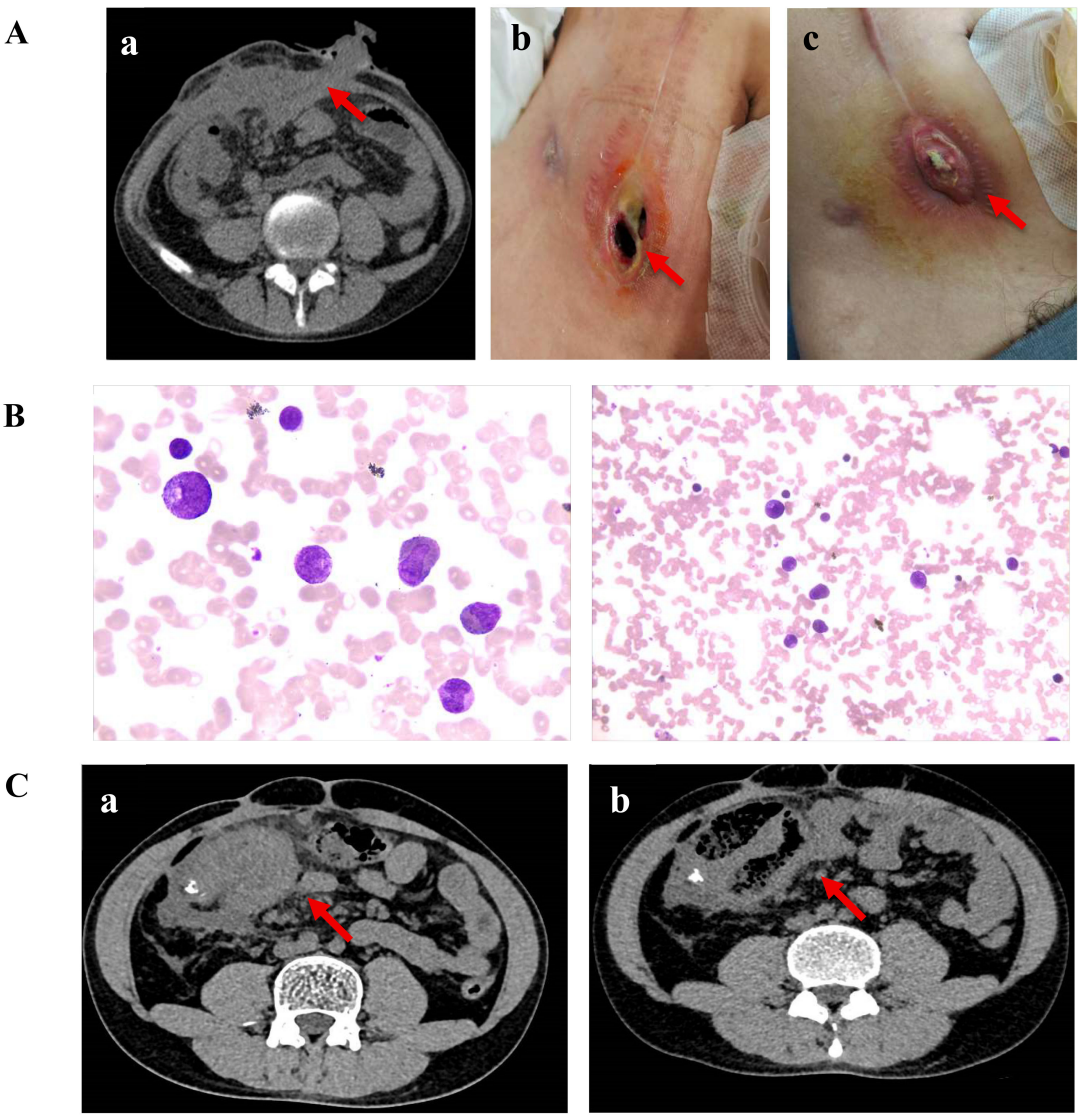
**(A)** (a) Postoperative CT imaging, (b) Postoperative wound appearance, with the arrow indicating an associated sinus tract in the right lower quadrant, (c) Wound appearance after treatment, showing substantial wound healing. **(B)** Bone marrow examination demonstrates granulocytic maturation arrest, characterized by an increased proportion of myeloblasts and promyelocytes and a marked reduction in mature granulocytes. **(C)** The two images show a comparative CT examination: (a) CT scan obtained before treatment with efbemalenograstim alfa, showing significant intestinal wall edema, (b) CT scan after 2 months of using efbemalenograstim alfa, showing marked resolution of the edema.

Postoperatively, the patient developed fever, persistent neutropenia, poor wound healing with suspected sinus tract formation (**Figures 3A**-b), localized skin infection, and anemia. Routine bacterial cultures of wound secretions were showed no growth. T-SPOT.TB testing and TB-DNA PCR analysis of the right colonic specimen were negative. Postoperative CT findings are shown in **Figure 3A**-a. Given these issues, a multidisciplinary consultation was conducted, and symptomatic management was initiated according to the recommendations. Hematology specialists recommended further evaluation, including bone marrow testing and biopsy, cytogenetic analysis, and a 31-gene eosinophil-related panel. However, the family refused further testing of the bone marrow, as multiple prior assessments at outside hospitals already showed reduced granulocytic proliferation and a markedly decreased proportion of late granulocyte precursors.

The patient received comprehensive supportive treatment, including broad-spectrum antibiotics, red blood cell transfusions, repeated wound care, and vacuum sealing drainage (VSD), recombinant human granulocyte-macrophage colony-stimulating factor (rhGM-CSF). Notably, following initiation of rhGM-CSF (200 μg/dose irregularly), the ANCs increased to above 1×10^9^/L, which correlated with improved wound healing and resolution of anastomotic edema (**Figure 3A**-c). The patient’s condition was stable, and he was subsequently discharged.

After discharge, the patient continued to experience intermittent abdominal pain, recurrent incision infections with purulent discharge, and persistent neutropenia, necessitating repeated hospital admissions. The patient was readmitted to our hospital. Following a comprehensive evaluation, the patient underwent a second surgery, including colectomy, stoma reversal, and adhesiolysis. Postoperatively, the patient remained severely neutropenic, prompting multidisciplinary consultations with hematology and gastroenterology teams.

Given the persistent and unexplained severe neutropenia, a congenital etiology was strongly suspected, and genetic testing was pursued. Whole-exome sequencing (WES) was performed on genomic DNA obtained from peripheral blood and identified a heterozygous missense variant in *ELANE* (NM_001972.4), c.242G>C (p.Arg81Pro), located in exon 3. This variant has previously been reported in patients with *ELANE*-associated neutropenia (11). According to the American College of Medical Genetics and Genomics (ACMG) guidelines, this variant was classified as likely pathogenic. ClinVar also lists the variant as likely pathogenic. The variant has been associated with autosomal dominant *ELANE*-related neutropenia, including SCN and CyN (3), and was not present in the 1000 Genomes Project, ESP, or ExAC databases. In silico prediction tools yielded mixed results, with PolyPhen-2 predicting a possibly damaging effect and SIFT predicting the variant to be tolerated. The genetic finding was highly consistent with persistent severe neutropenia, recurrent infections, and bone marrow evidence of granulocytic maturation arrest.

Subsequently, the patient was readmitted to our hospital due to intermittent abdominal pain and neutropenia, and a bone marrow examination was performed. The results demonstrated decreased granulocytic proliferation, with an increased proportion of myeloblasts and promyelocytes, and late granulocytic precursors were scarcely observed (**Figure 3B**), consistent with maturation arrest. Integrating the *ELANE* mutation, bone marrow findings, and the patient’s longitudinal laboratory data, a diagnosis of SCN was established. The gastrointestinal manifestations were considered to be strongly associated with chronic neutropenia and were most consistent with NE.

The patient received scheduled treatment with efbemalenograstim alfa (a long-acting G-CSF agent). Follow-up CT after 2 months showed substantial resolution of bowel wall edema (**Figure 3C**), with concurrent improvement in clinical symptoms. The patient tolerated the treatment well. At the time of writing (6 months later), the patient’s condition is stable, with no recurrent intestinal symptoms, severe infections, or further surgical complications. Close follow-up is maintained to properly manage further neutropenic episodes.

## 3 Discussion

SCN is a rare disorder marked by chronic, severe neutropenia and recurrent infections from early childhood (1). The present patient had a long-standing history of recurrent infections since infancy, which in retrospect strongly suggested an underlying congenital immunodeficiency. However, the diagnosis of SCN was not confirmed until adulthood, highlighting the diagnostic challenges. It also reveals the possibility of delayed recognition of this disease. The diagnosis was ultimately established through genetic confirmation of an underlying *ELANE*-associated neutropenia. In clinical practice, the diagnosis of SCN is based on clinical features (especially a history of recurrent infections from early life), persistently low ANCs (below 0.5×10^9^/L), bone marrow examination (showing maturation arrest of granulopoiesis), and genetic analyses (which confirm the diagnosis). Immunological analyses may also be performed to rule out other causes of neutropenia (1).

In this case, WES identified a heterozygous *ELANE* c.242G>C (p.Arg81Pro) variant, which was classified as likely pathogenic according to ACMG criteria and ClinVar records. *ELANE* mutations are the most common genetic cause of SCN and account for more than half of genetically characterized cases (1, 3). *ELANE*-associated neutropenia demonstrates substantial phenotypic heterogeneity, ranging from CyN to SCN. Previous studies have shown that even the same *ELANE* variant may be associated with different clinical severity, suggesting the influence of additional genetic or environmental factors on disease expression (3). The c.242G>C (p.Arg81Pro) variant has previously been reported in *ELANE*-associated neutropenia (11). The clinical phenotype in our patient, particularly the severe neutropenia and recurrent infections from early childhood, was consistent with previous reports of patients carrying this variant (11). The diagnosis was further supported by characteristic bone marrow findings demonstrating granulocytic maturation arrest.

CyN was considered in the differential diagnosis because it is another disorder associated with *ELANE* mutations. CyN is characterized by periodic oscillations of neutrophil counts, typically occurring every 21 days, with intermittent recovery of neutrophil levels between episodes (3). However, our patient exhibited persistent severe neutropenia during hospitalization, without evidence of cyclic fluctuations. The presence of granulocytic maturation arrest on bone marrow examination, together with recurrent infections, was consistent with SCN rather than CyN. A further diagnostic challenge in this case was the differential diagnosis of the gastrointestinal manifestations. Based on the patient’s previous diagnosis of UC, together with abdominal pain and postoperative histopathological findings, UC was initially considered. However, the histopathological examination showed deep ulceration extending into the muscularis propria. This finding was not entirely typical of classical UC, which is characterized by superficial mucosal inflammation, and prompted consideration of alternative diagnoses. The patient presented with recurrent oral ulcers and a history of anal fistula. This raised the possibility of Crohn’s disease as a diagnosis. In addition, Behçet’s syndrome was considered in the differential diagnosis because of the oral ulcers. However, the absence of other characteristic systemic manifestations and the presence of persistent severe neutropenia made these diagnoses less likely. Furthermore, considering the patient’s history of immunodeficiency, recurrent infections, and chronic neutropenia, monogenic IBD was also considered. Intestinal tuberculosis was also considered because of the ileocecal involvement, but both T-SPOT.TB and TB-DNA testing of the right colonic tissue were negative, providing no supportive evidence for this diagnosis. Ultimately, recurrent infections, long-standing neutropenia, identification of an *ELANE* mutation, and bone marrow findings demonstrating granulocytic maturation arrest supported SCN as the underlying hematologic disorder, providing an alternative explanation for the gastrointestinal manifestations. Although the initial histopathological interpretation favored UC, subsequent clinicopathological reassessment in the setting of chronic severe neutropenia suggested that these findings were not entirely specific for classical UC. Considering the chronic neutropenia, recurrent infections, bowel wall thickening on imaging, and the clinical response to granulocyte-stimulating therapy, the gastrointestinal manifestations were considered most consistent with NE secondary to SCN.

NE is a life-threatening digestive complication that typically occurs in immunocompromised patients, particularly those with profound neutropenia (12, 13). A prolonged period of neutropenia has been recognized as a major risk factor for the development of NE. It is also associated with increased mortality. Generally, NE has a poor prognosis. The average mortality rate is about 40–50% (7, 14). The pathogenesis of NE is not completely understood, but it is thought to result from intestinal mucosal injury in neutropenic patients, which can cause intestinal inflammation, necrosis, and potential perforation (8, 9). Clinically, the diagnosis of NE remains challenging due to a lack of specific criteria. In clinical practice, NE diagnosis is usually based on the presence of neutropenia(ANC<0.5 × 10^9^/L), fever, abdominal pain, bowel wall thickening >4 mm on imaging (CT is preferred to plain abdominal films and ultrasonography), and exclusion of other conditions, including Clostridium difficile-associated colitis, graft-versus-host disease(GVHD), and other abdominal syndromes (15, 16). In this patient, chronic neutropenia related to SCN likely impaired mucosal immune defense, facilitating bacterial invasion and intestinal inflammation, and eventually leading to NE. This case suggests that NE may be an underrecognized complication of congenital neutropenia. Overall, the gastrointestinal findings were most consistent with NE associated with SCN, rather than primary IBD.

Management of this patient was complex and required a multidisciplinary approach. Initial management included broad-spectrum antibiotics, surgical intervention for intestinal obstruction and supportive care. In this case, rhGM-CSF was used empirically before the confirmation of the diagnosis of SCN, resulting in clinical improvement, particularly in terms of wound healing and resolution of inflammatory edema. Currently, G-CSF remains the first-line therapy for SCN, and hematopoietic stem cell transplantation (HSCT) is an important radical and alternative treatment for patients who do not respond to G-CSF treatment, those with mutations in the *CSF3R* gene, or those who develop myelodysplastic syndrome or acute leukemia (1, 17). In contrast, the therapeutic management of NE has not been standardized to date due to the dearth of high-level evidence studies. Generally, the management of NE is largely supportive, including early antibiotics, bowel rest, blood product support, and hematopoietic growth factor treatment, while surgical interventions are still debated and may be necessary for patients who do not respond to conservative management or who experience complications such as abscess formation, significant bowel necrosis (7, 13). Following identification of *ELANE*-related SCN, the subsequent therapy of this patient was transitioned to efbemalenograstim alfa, resulting in sustained clinical stability.

Several limitations should be acknowledged. Parental genetic testing was not performed, which precluded further assessment of the inheritance pattern of the identified *ELANE* variant. The diagnosis of congenital neutropenia was only established after surgical intervention, as the underlying cause of the persistent neutropenia had not been fully recognized at the time of treatment. In addition, distinguishing NE from IBD in patients with chronic neutropenia remains challenging, particularly when gastrointestinal manifestations are prominent. Finally, the retrospective histopathological reassessment was not formally documented in a pathology report and should therefore be interpreted with caution.

This case highlights the value of long-term follow-up, genetic evaluation, and multidisciplinary reassessment in patients with persistent neutropenia presenting with gastrointestinal disease. Earlier identification of the underlying congenital neutropenia may have allowed more appropriate management and a more timely diagnosis.

## 4 Conclusion

SCN is a rare and often underrecognized disorder that may present with life-threatening complications such as NE. This case represents a rare presentation of *ELANE*-related SCN associated with NE. Our findings emphasize the importance of considering congenital neutropenia in patients with unexplained gastrointestinal symptoms and persistent neutropenia. Early recognition and management are essential to prevent misdiagnosis and improve clinical outcomes.

## Data Availability

The original contributions presented in the study are included in the article/supplementary material. Further inquiries can be directed to the corresponding authors.
